# Sports team participation in high school and adolescent and young adult depression and anxiety across sex, race/ethnicity, and socioeconomic status

**DOI:** 10.1111/jora.70246

**Published:** 2026-08-07

**Authors:** Malya Hirshkowitz, Lenna Nepomnyaschy

**Affiliations:** ^1^ School of Social Work Rutgers University New Brunswick New Jersey USA

**Keywords:** adolescent anxiety, adolescent depression, sports team participation, young adult anxiety, young adult depression

## Abstract

Poor mental health among adolescents and young adults in the United States today is a critical concern. Sports team participation, combining physical activity and social belonging, may be an effective component of interventions to improve mental health among this population. This study examines both short and long‐term associations between sports team participation in adolescence and anxiety and depression from adolescence to young adulthood, using the Future of Families and Child Wellbeing Study, a population‐based longitudinal birth cohort study of urban US children. In this sample of 2766 young adults (52% female, *M*_age_ = 22.3), controlling for a rich set of child and family characteristics, including prior mental health, we found that frequent sports participation in adolescence (33% of the sample), compared to none, was associated with substantially lower depression and anxiety symptoms among all adolescents. We also found that sustained sports participation in adolescence was associated with a lower likelihood of depression in young adulthood, but only for young adult males. Exploring heterogeneity in associations further by race/ethnicity and socioeconomic status, the strongest associations were found among young adult males who are non‐Hispanic white and Latino, and from higher socioeconomic backgrounds. These results suggest sustained sports team participation throughout adolescence may be a valuable intervention component for improving mental health among adolescents and young adults.

## INTRODUCTION

Depression and anxiety are pervasive and potentially life‐threatening concerns among adolescents and young adults in the United States. In 2024, 2.8 million adolescents (aged 12–17) reported depression that significantly impaired their functioning, and 3 million reported suicidal ideation (Reinert et al., 2025). Depression rates among adolescents and young adults have substantially increased in the past decade (Brody & Hughes, 2025). Rates among young adults have more than doubled from 13% in 2017 to nearly 27% in 2025 (Witters, 2025). Among individuals from low‐socioeconomic households, current reports of depression are close to 35% (Witters, 2025). Rates of anxiety have also risen dramatically over the last decade, with greatest increases found among young adults, aged 18–25 (Goodwin et al., 2020). While estimates differ, studies have found between 27% and 40% of 18 to 29‐year‐olds reporting symptoms of anxiety, the highest among all adult age groups (Srygley et al., 2023; Terlizzi & Zablotsky, 2024). Depression and anxiety are associated with numerous negative outcomes across the life course, including low academic achievement, substance use, sleep and appetite disturbances, reduced quality of life, and greater mortality and suicidal behaviors (Casares et al., 2024; Goodwin et al., 2020; Nzoma & Shaw, 2024).

Despite the high prevalence of these conditions, mental health treatment access and uptake in the United States are alarmingly low. Most adolescents experiencing mental health problems do not pursue or obtain treatment, either in the form of psychotherapy or pharmacological treatment (National Institute of Mental Health (NIH), 2023). In 2022–2023, over 50% of youth with major depression did not receive any form of clinical intervention or treatment (Reinert et al., 2025). Among the small proportion who do receive treatment, the efficacy of these treatments varies substantially (National Institute of Mental Health (NIH), 2023). Additionally, the most commonly prescribed medications—Serotonin‐specific reuptake inhibitors (SSRIs), Benzodiazepines, and Serotonin and norepinephrine reuptake inhibitors (SNRIs)—have many unpleasant side effects (e.g., agitation, headache, diarrhea, nausea, dizziness, sweating, sexual dysfunction, hypomania). Most dangerously, all SSRIs and SNRIs warn of increased risk of suicidal ideation, particularly among youth and young adults (FDA, 2018; Nzoma & Shaw, 2024). Therefore, there is an unmet need for accessible supplementary approaches to address mental health problems and improve mental health outcomes for these populations. In this study, we examine the associations of participation in team sports during adolescence with depression and anxiety in adolescence, whether these associations extend into early adulthood, and whether these associations differ by youth sex, race/ethnicity, and socioeconomic status.

### Conceptual framework

Bronfenbrenner's Ecological Systems theory provides a useful conceptual framework with which to understand how sports participation might impact mental health. The theory describes a nested system where an individual is at the center surrounded by increasingly broader layers of influence, often depicted as concentric circles (Bronfenbrenner, 1977). The theory illustrates the interactions and interrelationships that influence an individual. Building on this framework, Eime et al. (2013) developed the conceptual model of Health through Sport, which explicitly describes the relationships between factors that drive sport participation and the psychological and social health benefits associated with this participation. Their model illustrates the interactions and interrelationships between physical, social, and psychological factors.

Building on the “Health through Sport” conceptual model created by Eime et al. (2013), a narrative review by Martín‐Rodríguez et al. (2024) comprehensively examines how participation in sports may offer another method to improve youth and young adult mental health. The authors conceptualize sport not simply as physical activity (PA), but as a multilevel mechanism influencing mental health through interconnected psychological, social, and neurobiological pathways. They propose that PA serves as a potential tool for emotional regulation, stress reduction, resilience‐building, social connectedness, and treatment of mental health conditions. PA can trigger neurochemical and neurophysiological changes, increasing neurogenesis and synaptic functioning, which is linked to improvements in memory, learning, brain plasticity, and protection against cognitive decline.

Regular PA is well‐documented to have mental and physical health benefits, including the mitigation of depression and anxiety (Aguiar et al., 2014; Aguiar & Latini, 2018; Alderman et al., 2024; Blumenthal & Rozanski, 2023; Ma et al., 2024; Sharp et al., 2020). PA alters physiological and biochemical reactivity to stress and is associated with improved sleep, mood, and body image (Aguiar et al., 2014; Recchia et al., 2023). Furthermore, social connectedness acquired through experiencing social support, group membership, and social identity formation is associated with numerous positive mental and physical health outcomes. Social identification with one or more groups fosters a sense of purpose and often bolsters self‐esteem (Bang et al., 2024; Dingle et al., 2021; Haslam et al., 2022; Jetten et al., 2022; Leaper, 2011; Sani et al., 2012). Participation in team sports uniquely combines the benefits of PA and social connectedness and may potentially be an additional therapeutic tool for improving mental health among adolescents and young adults (Barchi et al., 2022; Harandi et al., 2017; Martín‐Rodríguez et al., 2024; Zuckerman et al., 2021).

### Associations of sports participation and mental health

A large body of work has documented associations between sports participation and lower levels of depression, anxiety, and other mental health problems in adolescence and young adulthood (Bang et al., 2024; Graupensperger et al., 2021; Hoffmann et al., 2022; Khan et al., 2023; Massey et al., 2024; Murray et al., 2021; Panza et al., 2020; Ramer et al., 2025; Zuckerman et al., 2021), including two recent meta‐analyses and a narrative review (Martín‐Rodríguez et al., 2024; Panza et al., 2020; Zuckerman et al., 2021). However, most of these studies, based on cross‐sectional data relying on contemporaneous measures of sports and mental health outcomes, present substantial challenges to assessing these potentially protective effects. The most serious challenges stem from the difficulty in accounting for unobserved factors between youth who participate and do not participate in sports, which may also contribute to mental health (e.g., prior mental health problems, socioeconomic status, family factors). Addressing these challenges calls for incorporating longitudinal analyses and including a robust set of relevant control variables.

Another challenge is distinguishing between individual and team sports, which some studies have done, finding stronger evidence of mental health benefits for participation in team compared to individual sports (Agans & Geldhof, 2012; Eime et al., 2013; Graupensperger et al., 2021). These differences may be particularly relevant for girls, as Graupensperger et al. (2021) found that team, but not individual, sports participation was associated with lower depression for girls, while for boys, both team and individual sports participation were beneficial.

While several prior studies used longitudinal data, measuring sports participation prior to mental health outcomes (Bang et al., 2024; Graupensperger et al., 2021; Khan et al., 2023), only two studies focused specifically on team sports participation and explicitly addressed the potential bidirectionality and unobserved differences between individuals (Graupensperger et al., 2021; Vella et al., 2017). Both studies took advantage of cross‐lagged panel models using longitudinal nationally representative Australian data but found somewhat conflicting results. First, team sports participation in early adolescence (12–13 years old) was associated with fewer internalizing behavior problems at age 14 and inversely higher levels of internalizing behavior problems at age 12 predicted lower team sports participation at 14 (Vella et al., 2017). Second, team sports participation in early adolescence was associated with fewer depressive and anxious symptoms at 14 and 16, but there were no such bidirectional effects for these outcomes (Graupensperger et al., 2021). Importantly, neither of these studies examined whether these protective effects extended into early adulthood.

Three other recent studies investigated whether the benefits of sports participation in adolescence extend into adulthood, with inconsistent results (Easterlin et al., 2019; Murray et al., 2021; Ramer et al., 2025). In one study, sports participation (without distinguishing between team or other sports) in high school (at ages 15 and 18) was associated with better mental health at age 26, but only for those who participated at both time points, not for those who stopped by age 18 (Ramer et al., 2025). In another study, sustained team sports participation from adolescence through young adulthood was associated with less stress and better coping, but not for those who stopped participating, and no protective associations were found for depression, even among sustainers (Murray et al., 2021). Finally, the third study found team sports participation in adolescence was associated with lower odds of depression and anxiety in adulthood (ages 24–32); however, this study focused only on youth who reported adverse childhood experiences (Easterlin et al., 2019). It is also important to note that some research documents potential negative impacts of sports participation, such as disordered eating, substance use, injury‐related distress, and, among some groups of male athletes, higher prevalence of violent sexual behaviors (Lütkewitte, 2023; Marchi et al., 2024; Murray et al., 2021; Staśkiewicz‐Bartecka et al., 2024).

### Differences by sex, race/ethnicity, and socioeconomic status

Sport functions as a highly visible social institution that can reinforce dominant gender norms, often privileging ideals associated with hegemonic masculinity (e.g., strength, competitiveness, and dominance) while marginalizing femininity (Birrell, 2000; Connell, 1987, 2020). As a result, girls and women participating in sport may encounter gender‐based discrimination and other systemic minority stressors that reflect broader societal power structures. According to Minority Stress Theory, exposure to these stressors can require ongoing psychological adaptation and may shape female's experiences and well‐being within sport environments (Meyer, 2003). Furthermore, female adolescents are nearly twice as likely as males to be diagnosed with depression and are more likely to report anxiety symptoms (Bang et al., 2024; Kajastus et al., 2024; Wilson & Dumornay, 2022). Additionally, compared to male adolescents, females have lower rates of team sports participation and are more likely to drop out as they approach young adulthood (Gómez‐Baya et al., 2020; Graupensperger et al., 2021; Khan et al., 2022, 2023; Ramer et al., 2025).

Many studies, but not all, have examined whether the protective effects of team sports participation for mental health outcomes differ by sex, and these results have also been inconsistent. A number of studies find stronger protective effects of team sports for males (Bang et al., 2024; Khan et al., 2023), including the meta‐analysis (Panza et al., 2020). Others found stronger effects for females (Graupensperger et al., 2021), and some found no statistical differences by sex (Easterlin et al., 2019; Hoffmann et al., 2022; Vella et al., 2017). However, in stratified analyses, Easterlin et al. (2019) found significant associations between sport participation and both depression and anxiety among males, whereas among females, significant associations were observed for anxiety only.

Next, a large body of research on the Social Determinants of Health demonstrates that non‐medical social and structural factors play a critical role in shaping health outcomes (US DHHS, n.d.). Race/ethnicity, as a marker of racism and racial residential segregation, and socioeconomic status are considered fundamental causes of health in the United States (Dwyer‐Lindgren et al., 2024) and are likely relevant factors for differences in adolescent and young adult well‐being. Because most studies in the United States have not been based on national data and the most thorough and recent studies are based on Australian data, there is little evidence regarding whether the benefits of team sports participation differ across key socio‐demographic factors, including race/ethnicity and socioeconomic status (Link & Phelan, 1995; Phelan & Link, 2015; Williams & Collins, 2001).

Intersectionality theory provides an important framework for understanding why the relationship between sport participation and mental health may differ across gender, class, and racial and ethnic groups. Intersectionality posits that discrimination is often experienced through multiple, overlapping systems of oppression, meaning that individuals may encounter marginalization not only based on gender, race, or their socioeconomic class alone but through their combined effects (Crenshaw, 1997). Within sport contexts, which often reflect broader societal inequalities, Black women and other women of color may experience both racial and gender marginalization, exposing them to unique stressors beyond the typical athletic demands faced by all athletes (Simien et al., 2019). For example, Black female collegiate athletes may experience heightened anxiety due to the psychological strain associated with racial and gender discrimination. More broadly, women of color are disproportionately affected by discrimination and marginalization in society, which can negatively impact mental and social well‐being (Ojemaye et al., 2024; Perry et al., 2013). These dynamics point to the importance of examining the effects of sport participation on depression and anxiety across these groups to better capture potential intersectional experiences.

### The current study

The current study comprehensively examines the association between sports team participation in adolescence and mental health outcomes in the United States. First, we estimate associations between sports team participation in adolescence and depressive and anxious symptoms in adolescence. Next, we examine whether any identified benefits extend into young adulthood, and finally, whether these associations differ by sex, race/ethnicity, and socioeconomic status. We take advantage of a population‐based, longitudinal, economically, racially, and ethnically diverse sample of 2766 young adults in the United States. We address potential selection into sports participation by including a comprehensive set of control variables, including, importantly, adolescents' mental health prior to high school (Easterlin et al., 2019). We hypothesize that more frequent participation in sports teams will be associated with fewer depressive and anxious symptoms in adolescence; however, our hypotheses regarding whether these benefits extend into adulthood and how they differ across socio‐demographic groups are ambiguous, given mixed or no evidence from prior research and the exploratory nature of these subgroup analyses.

## MATERIALS AND METHODS

### Data

This study uses secondary data from the Future of Families and Child Wellbeing Study (FFCWS), the longest‐running birth cohort study in the United States, which has been following 4898 children born between 1998 and 2000 in large US cities (populations of 200,000 or more). The FFCWS oversampled births to unmarried mothers by a ratio of 3 to 1, and the data are representative of all such births at that time. Mothers were interviewed at the birth of the focal child and were followed up at child ages 1, 3, 5, 9, 15, and 22, and children were interviewed at ages 9, 15, and 22 (James et al., 2021; Princeton University, 2025; Reichman et al., 2001).

### Sample

The current study examines adolescent sports team participation and mental health outcomes when youth were approximately 15 years of age (*M*_age_ = 15.6, SD = 0.75), from 2014 to 2017, and when youth were approximately 22 years of age (*M*_age_ = 22.3, SD = 0.52), from 2020 to 2024. Our analyses are based on youth interviewed at both the year‐15 and year‐22 survey waves (*N* = 2807, approximately 57% of the full baseline sample) and those who had no missing data on the self‐reported measures of depression at years‐15 and 22 (*N* = 2766, almost 99% of those interviewed at years‐15 and 22). All missing data on covariates were addressed using Full Information Maximum Likelihood (FIML) models within the structural equation modeling suite in Stata 18 (StataCorp, 2021).

### Measures

#### Sports team participation

The primary independent variables of interest are youths' self‐reports of how often they spent time on athletic or sports teams during the past school year at year‐15 and while they were in high school, asked retrospectively at year‐22. At year‐15, the measure is based on a three‐level categorical variable of: (1) never; (2) sometimes (less than once/month, at least once/month, or once/week); or (3) often (several times/week). For year‐22 analyses, we created an additional three‐level categorical variable combining youths' reports of participation across the two periods: (1) report of never participating in both periods; (2) report of sometimes participating in either period or an inconsistent report across periods; or (3) report of often participating in both periods.

#### Adolescent and young adult depression and anxiety

Adolescent self‐reported depression at year‐15 is derived from the Center for Epidemiologic Studies Depression Scale (CES‐D) (Radloff, 1977), as used in the National Longitudinal Study of Adolescent Health (Dennis et al., 2022), based on five items from the original 20‐item CES‐D: *(1) I feel I cannot shake off the blues, even with help from my family and my friends, (2) I feel sad, (3) I feel happy, (4) I feel life is not worth living, and (5) I feel depressed*. Possible responses, ranging from 1 = strongly disagree to 4 = strongly agree, were averaged across the five items with higher scores reflecting a higher presence of depressive symptoms (α = 0.76). At year‐22, the dichotomous measure of Young Adult Depression is derived from the Composite International Diagnostic Interview–Short Form (CIDI‐SF) (Kessler et al., 1998). Respondents are asked about feelings of dysphoria or anhedonia in the past year that lasted for 2 weeks or more and, if so, whether the symptoms lasted most of the day and occurred every day of the two‐week period. If so, they were asked more specific questions about (1) losing interest, (2) feeling tired, (3) change in weight, (4) trouble sleeping, (5) trouble concentrating, (6) feeling worthless, and (7) thinking about death. The dichotomous measure uses the liberal scale criteria, requiring the respondent to report 2 weeks of depressive symptoms over at least half the day. Respondents are classified as probable cases or probable non‐cases.

Adolescents' self‐reported anxiety at year‐15 is derived from the Brief Symptom Inventory 18 (BSI 18) (Derogatis, 2001). Originally an 18‐item assessment of psychological distress, six items are modified to create the anxiety subscale. The items include *(1) I have spells of terror or panic, (2) I feel tense or keyed up, (3) I get suddenly scared for no reason, (4) I feel nervous or shaky inside, (5) I feel fearful, and (6) I feel so restless I can't sit still*. Possible responses, ranging from 1 = strongly disagree to 4 = strongly agree, were averaged across the six items with higher scores reflecting a higher presence of anxious symptoms (α = 0.76). At year‐22, the dichotomous measure of young adult anxiety is derived from the CIDI‐SF for Generalized Anxiety Disorder (GAD) (Kessler et al., 1998). GAD is characterized by experiencing excessive worry or anxiety about multiple topics for 6 months or longer, occurring more days than not, and being difficult to control. Additional symptoms include feeling tense or on edge, irritability, restlessness, trouble falling asleep, fatigue, difficulty focusing, and muscle tension or aches. The dichotomous measure is classified as probable cases or probable non‐cases.

#### Covariates

Analyses include a rich set of child, parent, and family characteristics measured at different survey waves that may be associated with both adolescent team sports participation and mental health (Gore et al., 2001). First, we include a binary indicator of sex assigned at birth (the only measure available until year‐22), youths' age in years at the years‐15 and 22 surveys, and youths' self‐reported race/ethnicity (non‐Hispanic White, non‐Hispanic Black/African American, Hispanic/Latino, other) at year‐15. Next, we include whether the child was born at low birth weight (<2500 g), a strong marker of child health, and whether the child is the mother's firstborn. Characteristics of the mother at the time of the child's birth include whether she was married to the child's biological father (the primary sampling characteristic); education (<HS, HS, <HS); age (<21, 21–29, 30+); whether she is US‐born; and whether Medicaid paid for the child's birth, as a marker of low income at the child's birth. At the year‐1 survey, we include whether the mother met criteria for major depression, based on the Composite International Diagnostic Interview (CIDI)–Short Form (Kessler et al., 1998); and whether the child's biological father had a history of incarceration.

From the 9‐year survey, we include children's internalizing and externalizing behavior problems from the Child Behavior Checklist (CBCL) (Achenbach, 1999). Children's internalizing behavior problems are based on mothers' responses to 31 items from the anxious/depressed, withdrawn, and somatic symptom subscales, and children's externalizing behavior problems are based on mothers' responses to 35 items from the aggressive and rule‐breaking subscales. These scales are used as a lagged measure of adolescent mental health to address selection into sports participation if a child has mental health issues previously, as findings from prior work indicate that behavioral difficulties in grade 6 are associated with lower sport participation through high school (Ramer et al., 2025). Also from the year‐9 survey, we include household and contextual variables, including: the child's living arrangement (with both biological parents, with mother and mother's new partner, with unpartnered single mother, and other living arrangement); and mother's household income to poverty ratio categories, measured as household income from all sources, divided by the federal poverty thresholds (FPL) (<100%, 100–200%, or > 200% FPL). We also include the child's BMI, based on interviewer measured height and weight, and the frequency with which the mother reported playing sports or outdoor activities with the child in the past month (0 = not once to 4 = every day).

### Analytic strategy

First, we present descriptive results of all analysis variables for the full sample and by child's sex (binary, assigned at birth). Next, we estimate associations between sports team participation and adolescents' self‐reported depression and anxiety symptoms at year‐15; and young adults' self‐reported depression and anxiety at year‐22. At year‐15, outcome variables, depression and anxiety symptoms, are standardized (mean = 0, SD = 1) for ease of interpretation and for comparing coefficients across models (interpreted as changes in standard deviations). At year‐22, outcome variables are dichotomous indicators of “meets criteria” or “does not” for both depression and anxiety. We estimate linear full information maximum likelihood (FIML) models within the structural equation modeling (SEM) platform in Stata 18 (StataCorp, 2021) for the full sample at year‐15, and stratified by child sex (binary, assigned at birth). At year‐22, we further stratify models by adolescents' racial and ethnic identity by sex, and household poverty status at year‐9 (poor vs. not poor) by sex.

## RESULTS

### Descriptive results

Table 1 presents descriptive statistics of all analysis variables for the full sample and by sex (male/female assigned at birth). The sample of young adults was approximately 22 years old on average and was relatively equally divided between males and females (52%). At year‐15, 32% of adolescents reported never, 27% reported sometimes, and 41% reported often participating in team sports, representing a relatively active sample of adolescents. Using the combined year‐15 and year‐22 measure of sports participation, less than one‐quarter (24%) of young adults reported never participating in sports across the two periods, 43% reported sometimes or an inconsistent report across both periods, and 33% reported often participating across both periods. Girls and young women reported participating in team sports less frequently and less consistently in high school than their male counterparts across both measures.

**TABLE 1:** Descriptive statistics for analysis variables stratified by child sex.

	All	Boys/Men	Girls/Women	Sig diff
	Mean (SD) or proportion	Mean (SD) or proportion	Mean (SD) or proportion	
Mental health outcomes				
Depressive symptoms (1–4) at year 15	1.6 (0.60)	1.53 (0.55)	1.67 (0.63)	***
Anxiety symptoms (1–4) at year 15	1.8 (0.65)	1.76 (0.61)	1.86 (0.69)	***
Depressed (y/n) at year 22	0.39	0.29	0.48	***
Anxious (y/n) at year 22	0.13	0.1	0.17	***
Team sports participation in HS (year 15)				***
Never	0.32	0.26	0.38	
Sometimes	0.27	0.26	0.28	
Often	0.41	0.48	0.34	
Team sports participation in HS (year 15 and 22)				***
Never (both reports)	0.24	0.19	0.3	
Sometimes or inconsistent reports	0.43	0.42	0.43	
Often at both reports (sustainers)	0.33	0.39	0.27	
Control variables				
Internalizing behavior problems (year 9)	0.16	0.16	0.16	
Externalizing behavior problems (year 9)	0.18	0.19	0.16	***
Race/ethnicity (year 15)				*
White, non‐Hispanic	0.18	0.27	0.23	
Black/African American, non‐Hispanic	0.48	0.46	0.5	
Hispanic/Latino	0.27	0.27	0.27	
Other, non‐Hispanic	0.07	0.09	0.06	
Low birth weight	0.1	0.1	0.1	
First born	0.4	0.4	0.4	
Mother married at child's birth	0.25	0.27	0.24	
Mother's education at child's birth				*
Less than high school	0.31	0.28	0.32	
HS or equivalent	0.31	0.32	0.31	
Some college or tech	0.38	0.4	0.37	
Mother's age at child's birth				
Less than 21	0.34	0.33	0.35	
21–29	0.43	0.44	0.43	
30+	0.23	0.23	0.22	
Mother born in US	0.87	0.87	0.86	
Medicaid used to pay for child's birth	0.6	0.59	0.62	
Mother met depression criteria (year 1)	0.16	0.16	0.16	
Father ever incarcerated (year 1)	0.33	0.33	0.34	
Child's living arrangement (year 9)				
With both biological parents	0.39	0.46	0.45	
With mother and new partner	0.18	0.19	0.18	
With unpartnered single mother	0.36	0.35	0.37	
Other living arrangement	0.07	0.07	0.07	
Mother's household income (year 9)				***
<100% FPL	0.35	0.32	0.37	
100–200%	0.29	0.28	0.3	
>200%	0.36	0.4	0.33	
Child's BMI (year 9)	19.6 (4.7)	19.26 (4.6)	19.9 (4.9)	***
Frequency parents play sports w/ child (year 9) (1–5)	2.92 (1.05)	2.9 (1.1)	2.96 (1.1)	
Age at year 15 (14–19)	15.55 (0.74)	15.56 (0.73)	15.54 (0.72)	
Age at year 22 (21–26)	22.27 (0.52)	22.29 (0.51)	22.26 (0.49)	
*N* (% of sample)	2766 (100%)	1327 (48%)	1439 (52%)	

*Note*: Significance tests based on *t*‐tests for continuous and binary variables and chi‐square tests for categorical variables.

Abbreviations: BMI, body mass index; FPL, federal poverty line; HS, high school; SD, standard deviation; sig diff, statistically significant differences; y/n, yes/no.

* *p* < .05.

*** *p* < .001.

Adolescents had an average score of 1.6 (scale of 1–4, SD = 0.60) on the self‐reported depressive symptom scale and an average score of 1.8 (scale of 1–4, SD = 0.65) on the self‐reported anxiety scale. At year‐22, 39% of the young adults in the sample met criteria for depression, a proportion in line with recent reports of depression prevalence among all young adults (26.7%) and among those from lower‐socioeconomic backgrounds (35%) (Witters, 2025). The dichotomous measure of anxiety at year‐22 indicated that 13% of young adults met criteria for anxiety (lower than reported national averages of 18%–36%) (Making Caring Common, 2023; Terlizzi & Zablotsky, 2024). Female adolescents and young adults had significantly higher depression and anxiety scores and prevalence rates than their male counterparts across both time periods, in line with prior work (Bang et al., 2024; Kajastus et al., 2024; Wilson & Dumornay, 2022).

Reflecting the design of the FF study, which oversampled children born to unmarried parents (75% of sample) in urban areas, the analysis sample is more racially and ethnically diverse and of lower‐socioeconomic status compared with a national sample. Few of the covariates differed significantly between male and female adolescents.

### Multivariate results

Table 2 presents multivariate results for the associations of the frequency of sports participation at year‐15 with adolescents' self‐reported depressive (Panel A) and anxiety symptoms (Panel B) at year‐15 for the full sample and stratified by child sex. Both outcome variables (depressive and anxious symptoms) have been standardized (mean = 0, SD = 1). Models include all covariates discussed previously (Table 1), but only the sports participation coefficients (and t‐statistics) are presented. Results in Panel A indicate that frequent sports participation is associated with nearly one‐quarter of a standard deviation (−0.23 SD; *p* < .001) lower depressive symptom scores compared to youth who never participated, and these associations are nearly identical for both sexes (males: −0.25 SD; *p* < .001; females: −0.22 SD; *p* < .001). There is no difference between sometimes and never participating, for either group. Likewise in Panel B, frequent sports participation is associated with nearly one‐quarter of a standard deviation (−0.22 SD; *p* < .001) lower anxiety symptom scores compared to youth who never participate, with similar associations for both sexes (males: −0.19 SD; *p* < .01; females: −0.24 SD; *p* < .001). Again, there is no difference between sometimes and never participating. Post‐hoc interaction tests confirmed no statistically different associations between male and female adolescents for both outcomes.

**TABLE 2:** High school team sports participation and mental health outcomes at year‐15.

	All	Boys	Girls
**Panel A: Depressive symptoms (standardized)**			
Team sports participation in HS Year 15 (reference = never)			
Sometimes	−0.075	−0.081	−0.088
	(−1.54)	(−1.18)	(−1.30)
Often	−0.23***	−0.25***	−0.22***
	(−5.05)	(−4.02)	(−3.31)
N	2766	1327	1439
**Panel B: Anxious symptoms (standardized)**			
Team sports participation in HS Year 15 (reference = never)			
Sometimes	−0.060	0.020	−0.12
	(−1.22)	(0.28)	(−1.82)
Often	−0.22***	−0.19**	−0.24***
	(−4.89)	(−3.13)	(−3.60)
N	2766	1327	1439

*Note*: Figures are ordinary least squares (OLS) coefficients and (t‐statistics). Models include all previously described covariates.

Abbreviation: HS, high school.

** *p* < .01.

*** *p* < .001.

Table 3 presents multivariate results of the associations of the frequency and consistency of team sports participation in high school (combined across the years‐15 and 22 reports) with young adult self‐reported depression (Panel A) and anxiety (Panel B) at year‐22. In Panel A, young adults who reported often participating in team sports in high school consistently at years‐15 and 22 had 8.3 percentage points lower likelihood (−0.083; *p* < .01) of meeting criteria for depression compared to those who consistently reported never participating, translating to a 20% lower likelihood off the sample mean. In addition, those who reported less frequent or inconsistent participation across the two timepoints had 4.9 percentage points lower likelihood (−0.049; *p* < .05) of depression compared to those who consistently reported never participating. Stratified by sex, significant associations are observed only among males. Compared to those who consistently never participated in sports, males who consistently reported frequent participation had 18 percentage points lower likelihood of depression (−0.18; *p* < .001), translating to a 46% lower likelihood off the sample mean. Those who reported less frequent or inconsistent participation had 11 percentage points lower likelihood (−0.11; *p* < .01). Post‐hoc interaction tests confirmed these statistically significant differences in associations between male and female young adults (*p* < .01).

**TABLE 3:** High school team sports participation and mental health outcomes at year‐22.

	All	Men	Women
**Panel A: Depressed (yes/no)**			
Team sports participation in HS at year 15 and 22 (reference = never at both periods)			
Sometimes or inconsistent reports	−0.049*	−0.11**	−0.013
	(−1.99)	(−3.13)	(−0.40)
Often at both reports (sustainers)	−0.083**	−0.18***	−0.011
	(−3.17)	(−4.88)	(−0.28)
N	2766	1327	1439
**Panel B: Anxious (yes/no)**			
Team sports participation in HS at year 15 and 22 (reference = never at both periods)			
Sometimes or inconsistent reports	−0.015	−0.062*	0.012
	(−0.77)	(−2.47)	(0.44)
Often at both reports (sustainers)	−0.010	−0.051*	0.0087
	(−0.50)	(−1.96)	(0.28)
N	2766	1327	1439

*Note*: Figures are ordinary least squares (OLS) coefficients and (t‐statistics). Models include all previously described covariates.

Abbreviation: HS, high school.

* *p* < .05.

** *p* < .01.

*** *p* < .001.

Results for Panel B in Table 3 reveal no statistically significant association between participating in sports in high school and 22‐year reports of anxiety in the full sample. However, when stratifying by sex, we do see protective associations, but only for males. Compared to males who consistently never participated, those who participated often (at both periods) and those who sometimes or inconsistently reported participating were 5 and 6 percentage points (*p* < .05) less likely to have anxiety, respectively. There were no statistically significant associations for females; however, post‐hoc interaction tests did not find these differences by child sex to be statistically significant; thus, these differences should be interpreted with caution.

Table 4 extends the previous results through exploratory subgroup analyses by investigating differences in associations by race/ethnicity and socioeconomic status. Results in Panel A indicate that participating in sports often in high school, compared to never, is associated with lower depression for non‐Hispanic white and Hispanic/Latino males, but much less so for non‐Hispanic Black males. Among females, protective associations of sports participation are only observed for non‐Hispanic white participants and are marginally significant (*p* < .10). We find that frequent sports participation, compared to never, is associated with higher probability of depression (11 percentage points) among Black female young adults (*p* < .05). However, post‐hoc interactions did not find these differences to be statistically significant.

**TABLE 4:** High school team sports participation and mental health outcomes at year 22 stratified by sex, race/ethnicity, and poverty status.

	Race/Ethnicity						Poverty status			
	Men			Women			Men		Women	
	Non‐Hispanic White	Non‐Hispanic Black	Hispanic/Latino	Non‐Hispanic White	Non‐Hispanic Black	Hispanic/Latino	Poor	Non‐poor	Poor	Non‐poor
**Panel A: Depressed (yes/no)**										
Team sports participation in HS Year 15 and 22 (reference = never at both periods)										
Sometimes or inconsistent reports	−0.12	−0.10^+^	−0.16*	0.017	−0.012	0.013	−0.088	−0.12**	0.072	−0.047
	(−1.49)	(−1.81)	(−2.03)	(0.22)	(−0.26)	(0.19)	(−1.32)	(−2.62)	(1.31)	(−1.07)
Often at both reports (sustainers)	−0.25**	−0.090	−0.25**	−0.17^+^	0.11*	−0.032	−0.072	−0.22***	0.12^+^	−0.091^+^
	(−2.95)	(−1.55)	(−3.18)	(−1.89)	(2.01)	(−0.42)	(−1.02)	(−4.67)	(1.81)	(−1.88)
N	246	598	308	259	693	331	391	818	478	831
**Panel B: Anxious (yes/no)**										
Team sports participation in HS Year 15 and 22 reference = never at both periods										
Sometimes or inconsistent reports	−0.0013	−0.085*	−0.039	−0.032	0.038	0.0021	0.011	−0.11**	0.047	−0.026
	(−0.02)	(−2.34)	(−0.65)	(−0.39)	(1.14)	(0.03)	(0.25)	(−3.26)	(1.14)	(−0.62)
Often at both reports (sustainers)	−0.068	−0.072^+^	−0.028	−0.14	0.091*	0.062	−0.030	−0.076*	0.073	−0.022
	(−1.17)	(−1.92)	(−0.43)	(−1.44)	(2.31)	(0.93)	(−0.62)	(−2.17)	(1.33)	(−0.49)
N	246	598	308	259	693	331	391	818	478	831

*Note*: Figures are ordinary least squares (OLS) coefficients and (t‐statistics). Models include all previously described covariates.

^+^ *p* < .10.

* *p* < .05.

** *p* < .01.

*** *p* < .001.

Stratification by socioeconomic status indicates significant protective associations only for non‐poor male (−0.22, *p* < .001) and female (−0.09, *p* < .10) young adults. On the contrary, we observe a higher likelihood of depression among poor female young adults who participated in sports often (0.12; *p* < .10) compared to those who did not. Post‐hoc interaction tests confirmed that differences between poor and non‐poor female young adults were statistically significant, but not between males.

Panel B examines these same associations for young adult reports of anxiety. Stratification by race/ethnicity indicates the opposite of that for depression among males. Participation in sports was only associated with lower anxiety for non‐Hispanic Black male young adults. But, similarly to depression, we find that for Black female young adults, frequent sports participation, compared to never, was associated with a higher probability (9 percentage points; *p* < .05) of anxiety. The difference in associations between non‐Hispanic Black and white female young adults was found to be statistically significant in post‐hoc interaction tests (*p* < .01), but no differences were found among males by racial/ethnic group. Stratifying by socioeconomic status, as for depression, revealed that sports participation is only protective for non‐poor male young adults, and this difference in associations was confirmed by statistically significant post‐hoc interaction tests (*p* < .05).

## DISCUSSION

Youth and young adult mental health is a serious concern, as the prevalence rates of depression and anxiety symptoms have increased tremendously in the United States particularly since the COVID‐19 pandemic (Terlizzi & Zablotsky, 2024). Traditional treatments such as psychotherapy and pharmacology often vary in terms of efficacy and may carry risks, including potential side effects like increased suicidal thoughts and behaviors (FDA, 2018; Nzoma & Shaw, 2024). Sports team participation, which brings together physical activity and social connections, factors that are associated with better mental and physical health among youth, may serve as a useful approach for mental health promotion. Our study aimed to comprehensively examine this question, exploring associations of sports team participation in high school with contemporaneous mental health outcomes in adolescence, exploring whether these associations extend into young adulthood, and investigating whether these associations differ by youth sex, race/ethnicity, and socioeconomic status. We take advantage of a large, population‐based longitudinal study based on a racially, ethnically, and socioeconomically diverse sample of US youth.

We find that between 30% and 40% of youth participated often in sports in high school and between one‐quarter and one‐third never participated. Importantly, in line with prior work, across measures and over time, we find consistently lower rates of sports participation and worse mental health among females. In our multivariate analyses, we control for a rich set of factors that may be associated with selection into sports participation and with mental health outcomes, including prior internalizing and externalizing behaviors when children were 9 years old, which have been found to be strongly predictive of sports participation in high school (Ramer et al., 2025; Vella et al., 2017). We find an overall strong negative association between frequent team sports participation in high school and depression and anxiety in adolescence, with effect sizes of nearly one‐quarter standard deviation lower depression and anxiety symptoms compared to those who never participated. These results are in line with much prior work finding that sports participation was strongly associated with lower levels of depression and anxiety in adolescence (Bang et al., 2024; Graupensperger et al., 2021; Hoffmann et al., 2022; Khan et al., 2023; Massey et al., 2024; Zuckerman et al., 2021), but inconsistent with others, such as the meta‐analysis (Panza et al., 2020), which found a weak effect size of this association. Importantly, we also find that these associations and magnitudes do not differ by adolescent sex (assigned at birth). Prior research has found conflicting results of sex differences. Some studies find stronger protective effects of team sports for males (Bang et al., 2024; Khan et al., 2023; Panza et al., 2020), others find stronger protective effects for females (Graupensperger et al., 2021), and some find no statistical differences by sex similarly to our results (Easterlin et al., 2019; Hoffmann et al., 2022; Vella et al., 2017).

Next, our analyses built on this prior work by exploring whether the benefits of sports team participation for adolescents' mental health extend into young adulthood (Easterlin et al., 2019; Murray et al., 2021; Ramer et al., 2025). We incorporate a unique measure of sports participation in high school, combining contemporaneous reports of participation at year‐15 and retrospective reports at year‐22. We test associations between consistent reports of frequent (often) participation, compared to consistent reports of never participating, with the probability of having depression and anxiety at year‐22. Here, our results present a greater level of variability and nuance. For both outcomes, compared to never participating, we find protective effects of consistent reports of frequent participation, but only for males, indicating that these beneficial effects of sports participation in adolescence do not extend into young adulthood for females. Nonetheless, the associations for males are quite strong, representing between 33% and 50% lower likelihood of anxiety and depression, respectively. In supplementary analyses (not shown), we estimate these models using a continuous measure of depression symptoms at year‐22 to make more valid comparisons to the measure of depression at year‐15. The pattern of results remains identical to the original binary depression measure, showing a protective association of sustained participation in team sports with depression for the full group of young adults, but only significant associations for male young adults.

Although several studies have examined sex differences in the relationship between sport participation and mental health outcomes, the underlying reasons for disparities in observed effects among females remain insufficiently explained (Bang et al., 2024; Graupensperger et al., 2021; Ramer et al., 2025). Girls and women not only exhibit higher rates of depressive and anxiety symptoms but also face compounded barriers to sport engagement compared to boys and men. These barriers often operate across multiple levels: intrapersonal factors such as low confidence and body image concerns; inter‐personal factors such as unsupportive family or peer attitudes; and structural factors such as time constraints, safety concerns, and financial costs (He et al., 2024). To address these persistent inequities, future research should prioritize understanding the mechanisms driving these gendered differences in sport participation and mental health outcomes. Mixed‐methods and qualitative approaches are especially valuable for uncovering the nuanced, context‐specific experiences of girls and young women that quantitative data alone may overlook.

Finally, we stratify models further to explore differences by sex and race/ethnicity, and sex and socioeconomic status. Here, for depression, we find protective associations of sports team participation primarily for white and Latino males; and for anxiety, for Black males. We find that sports participation is associated with worse mental health (for both anxiety and depression) for Black females. Additionally, for both outcomes, we find protective associations only for non‐poor young adults, particularly males, and some evidence of harmful associations for poor Black females. However, across these more nuanced subgroup analyses, we find few statistically significant interaction effects, as these analyses substantially reduce sample sizes and statistical power, pointing to the need for caution when considering these results. Nonetheless, these exploratory findings highlight the importance of considering how structural and contextual factors such as race and ethnicity, as markers of racism and segregation, and socioeconomic status may shape the mental health implications of sport involvement. Future research should prioritize examining these subgroups in both quantitative and qualitative work to better understand the mechanisms through which sport participation may differentially influence psychological well‐being.

Our findings pointing to the potentially harmful associations of sports participation with mental health, particularly for Black female young adults, are in line with some prior work. For example, some studies have identified increased risk of disordered eating and related mental health concerns (Marí‐Sanchis et al., 2022; Moore et al., 2022; Staśkiewicz‐Bartecka et al., 2024). Other areas of sport‐related research have examined the higher prevalence of violent sexual behaviors and harmful attitudes towards women among certain groups of male athletes (Lütkewitte, 2023; McMahon, 2007). Other research has linked team sport participation to greater substance use (Murray et al., 2021). Frequent occurrence of sport‐related injuries, especially for competitive and elite athletes, can lead to significant stress and problematic mental health symptoms (Putukian, 2022; Schinke et al., 2018). Some research has found sports participation to be associated with increased anxiety (Gabrys & Wontorczyk, 2023; Stracciolini et al., 2020), but primarily for individual (vs. team) sports and particularly for females (Bang et al., 2024; Hoffmann et al., 2022; Khan et al., 2022, 2023). Black female athletes of color may face all these same stressors while also being exposed to racial and gender discrimination due to their intersecting social identities, putting them further at risk of poor mental health outcomes (Ojemaye et al., 2024). These complex and nuanced findings certainly warrant further exploration.

Our results must be considered in the context of several limitations. First, as with all observational studies, we cannot infer causality. Although we included a rich set of potential confounders, especially a lagged measure of children's mental health (internalizing and externalizing behavior problems) from a prior wave, and took advantage of longitudinal data (measuring depression and anxiety several years after sports participation), there is still a possibility of unmeasured systematic differences between sport participants and non‐participants. For example, youth who possess greater athletic ability, social confidence, or prior positive sport experiences may be more likely to sustain sport team participation. These same characteristics may independently promote stronger mental health outcomes. As such, sustained team sport participation cannot be assumed to function as a universally beneficial intervention without consideration of team structure, inclusivity, and youth characteristics. Furthermore, mental health problems may discourage youth from engaging in sports, compounding the challenge of disentangling cause and effect. In the current study, we controlled for child mental health prior to our measure of sports participation, but we were not able to formally assess potential bidirectionality between sports and mental health, because sports participation was only measured during high school. Future research should consider experimental or quasi‐experimental approaches to address selection bias, potential bidirectionality, and to strengthen causal inference. Although the present study did not examine mechanisms, future intervention studies should also investigate potential pathways linking sports participation to mental health outcomes, such as enhanced social belonging, social support, or physical activity. Experimental designs that compare sports participation with other extracurricular activities (e.g., theater groups, clubs, choirs, or bands) could help distinguish the effects of sport‐specific factors from those attributable to social connectedness and belonging more broadly.

As with all longitudinal studies, the FFCWS experiences sample attrition over time. In analyses not shown, we compare the analytic sample to those excluded due to attrition from the study on baseline characteristics. Consistent with prior work (e.g., Teitler et al., 2003), we find the analytic sample to be more socioeconomically advantaged, supporting our inclusion of family socioeconomic covariates at the time of the child's birth. Thus, it is likely that participants who remain in the study are healthier than those who are lost to follow‐up. As a result, although the prevalence of depression in our analytic sample is already alarmingly high, the true population prevalence is likely to be even higher. However, recent work suggests that this attrition problem may not affect the associations of sports participation with youth mental health found in the current study (Saiepour et al., 2019).

Also, our study considers only anxious and depressive symptoms, a narrow set of outcomes. It is important to note that sports participation may be associated with a wide range of social, mental, and behavioral outcomes, both protective and risk‐inducing, that are beyond the scope of the present analyses. Additionally, the reliance on a single‐question measure of sports team participation frequency in the FFCWS data may limit the ability to capture the nuanced aspects of sports team involvement (e.g., level of competition, team dynamics, or the degree to which individuals identify with being an athlete). Expanding these measures in future research would provide a deeper understanding of how specific aspects of sports engagement may influence mental health outcomes. Nonetheless, this variable was asked explicitly as participation in team sports and thus may be a strength in comparison with prior studies which were less explicit (Panza et al., 2020), potentially reducing measurement error. Finally, although we include a comprehensive set of covariates, future research should also consider school‐ and community‐level factors such as resources, school climate, neighborhood safety, and exposure to violence. Perceived safety and social environment may shape participation patterns across diverse groups (Ghorbani, 2025). Future research could also benefit from exploring potential mechanisms of these associations (e.g., increased physical activity and sense of social belonging) to understand how this might be operating for all young adults and across relevant socio‐demographic groups.

## CONCLUSION

Despite these limitations, our findings contribute valuable knowledge that can inform policy recommendations and program development. The results provide a robust foundation for leveraging sports participation in initiatives designed to promote positive mental health outcomes among adolescents. These insights further emphasize the potential for sports team participation to serve as an additional component in treatment plans, prevention strategies, and interventions. Sport‐based intervention programs similarly demonstrate the potential for positive mental, physical, and social well‐being outcomes among youth (Hershow et al., 2015; Kaplan et al., 2015; Kaufman et al., 2013; Klemmer et al., 2025; Sutcliffe et al., 2024). However, our results also point to important differences in demographic groups as we found that sustained sports participation in adolescence was associated with a lower likelihood of depression in young adulthood, but only for young adult males. The strongest associations were found among young adult males who are non‐Hispanic white and Latino, and from higher socioeconomic backgrounds. These findings emphasize the importance of considering demographic differences when designing and implementing sport initiatives. Our findings contribute to important evidence for the implementation of innovative approaches to improve adolescent and young adult mental health in the United States.

## AUTHOR CONTRIBUTIONS

**Malya Hirshkowitz:** Conceptualization; methodology; formal analysis; data curation; writing – original draft; writing – review and editing. **Lenna Nepomnyaschy:** Methodology; formal analysis; data curation; writing – original draft; writing – review and editing.

## FUNDING INFORMATION

The authors have nothing to report.

## CONFLICT OF INTEREST STATEMENT

The authors declare no conflict of interest.

## ETHICS STATEMENT

The authors have nothing to report.

## CONSENT STATEMENT

The authors have nothing to report.

## Data Availability

This study used data from the Future of Families and Child Wellbeing Study (FFCWS), which are publicly available from the FFCWS site: https://ffcws.princeton.edu/.
